# Association between dynamic digital radiography findings and post-extubation respiratory deterioration: A retrospective exploratory analysis of a prospectively collected ICU cohort

**DOI:** 10.1371/journal.pone.0352029

**Published:** 2026-06-22

**Authors:** Satoshi Komatsu, Kazuki Nishida, Yoshitaka Hara, Naohide Kuriyama, Tomoyuki Nakamura

**Affiliations:** 1 Department of Anesthesiology and Critical Care Medicine, Fujita Health University School of Medicine, Aichi, Japan; 2 Department of Biostatistics, Kyoto University School of Public Health, Kyoto‌‌, Japan; Stamford Health System: Stamford Hospital, UNITED STATES OF AMERICA

## Abstract

**Introduction:**

Dynamic Digital Radiography (DDR) is a novel bedside imaging modality that enables real-time visualization of pulmonary motion with minimal radiation exposure. We evaluated whether DDR-derived parameters could assist in stratifying the risk of post-extubation respiratory deterioration in ICU patients.

**Methods:**

We analyzed a prospectively collected, single-center cohort of consecutive adults extubated after invasive mechanical ventilation between December 2024 and February 2025 (n = 56). Bedside DDR was performed immediately before and within 24 hours after extubation; respiratory deterioration was defined as any increase in oxygen supplementation before ICU discharge. We used logistic regression to address the initial objectives and added exploratory machine learning to explore predictive performance, with interpretability assessed using Shapley additive explanations (SHAP).

**Results:**

While no significant differences were found in directly measured DDR values between groups, age and pre-extubation respiratory rate differed significantly between patients with and without respiratory deterioration. In logistic regression analysis, post-extubation lung-area excursion was not significantly associated with respiratory deterioration after adjustment for age. In exploratory XGBoost/SHAP analysis, age, post-extubation respiratory rate, and post-extubation lung-area excursion showed relatively large model-based contributions, but these findings were interpreted as exploratory feature contributions rather than evidence of independent statistical association.

**Conclusions:**

In this exploratory ICU cohort, DDR-derived post-extubation lung-area excursion was not independently associated with respiratory deterioration after adjustment for age, but it showed a relatively large model-based contribution in exploratory XGBoost/SHAP analysis. DDR-derived dynamic information may provide complementary, hypothesis-generating information for post-extubation assessment. Larger prospective studies with external validation are required before DDR-derived indices can be considered clinically validated predictors.

## Introduction

Respiratory diseases are the most common cause for admission to the intensive care unit (ICU), accounting for approximately 40% of all cases [1]. Appropriate mechanical ventilation is essential in the management of such patients. Especially, deterioration in respiratory status after extubation is an important clinical issue, as it may lead to reintubation, prolonged ICU stay, increased healthcare costs, and higher mortality [2,3].

Accordingly, accurate assessment of readiness for extubation and early identification of patients at risk of post-extubation respiratory deterioration are critical to improving the quality of ICU care. Traditional assessment methods include respiratory support indices such as the Rapid Shallow Breathing Index (RSBI) and cough peak flow, clinical observations, and blood gas analysis [4]. However, these indicators are often influenced by the patient’s cooperation and the timing of evaluation, and are limited in their ability to dynamically and objectively assess pulmonary function and ventilation status.

Chest radiography and computed tomography (CT) can reveal structural abnormalities of the lungs, but real-time evaluation of respiratory-phase-specific lung dynamics is difficult. Moreover, CT may not be suitable for critically ill patients due to its high radiation exposure and the physical burden associated with patient transport [5].

**Fig 1:**
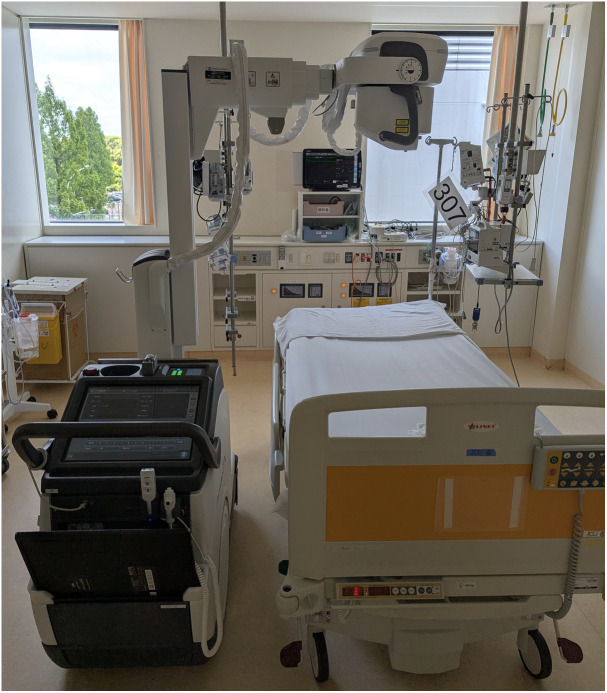
Portable dynamic digital radiography (DDR) system used for bedside imaging in ICU patients. The portable DDR system used in the present study for bedside assessment of respiratory motion in mechanically ventilated ICU patients. The system enables sequential low-dose chest radiographic imaging during tidal breathing and allows dynamic visualization of respiratory-related changes in lung motion and lung area at the bedside.

Dynamic Digital Radiography (DDR) is a novel X-ray technique that combines a high-speed flat panel detector with advanced image processing to visualize respiratory and circulatory motion in real time and with low radiation exposure [6] (Fig 1). In recent years, its application has been reported in airway obstruction assessment and diaphragmatic motion analysis [7,8]. With portable devices, DDR can also be performed at the bedside. Furthermore, dedicated analysis software such as KINOSIS allows for the automatic extraction of quantitative parameters including LV max area, LV min area, diaphragm excursion, pulmonary perfusion, and respiratory rate, enabling rapid, objective, and noninvasive evaluation with minimal operator dependence [9].

Nevertheless, few systematic studies have examined the utility of DDR in critically ill patients, and evidence is lacking regarding the association between DDR indices before and after extubation and post-extubation respiratory deterioration. In addition, the usefulness and interdependence of each DDR index in relation to other clinical factors for risk stratification remain unclear.

This study aimed to retrospectively evaluate whether DDR data obtained before and within 24 hours after extubation in adult ICU patients were associated with post-extubation respiratory deterioration, defined as an increased oxygen requirement.

## Methods

### Study design and ethics‌‌

This was a single-center cohort study of prospectively collected, consecutive ICU extubation cases conducted at Fujita Health University Hospital. We included consecutive adult patients who were already endotracheally intubated at ICU admission and subsequently underwent extubation between December 2024 and February 2025. Patients were excluded if they were aged under 18 years, did not consent to the use of their clinical data for research purposes, or could not undergo DDR before or within 24 hours after extubation. Given the rarity of bedside DDR during the study window, we included all eligible consecutive cases and did not set a fixed sample-size target.

The study was approved by the Ethics Committee of Fujita Health University (Approval No. HM25–097). All methods were performed in accordance with the relevant guidelines and regulations, including the Declaration of Helsinki and the institutional policies of Fujita Health University. Written informed consent was obtained from all participants or their legal guardians at ICU admission. An opt-out method was used to provide patients or their proxies with information about the study and to ensure an opportunity to decline participation. This study was conducted in accordance with the STROBE guidelines. Data for this study were accessed for research purposes on 15/07/2025.

### DDR imaging and parameter extraction

All patients underwent portable DDR (Shimadzu Corp., Japan) in the supine position immediately before and within 24 hours after extubation. The supine position was used to ensure feasibility in the ICU setting and to standardize imaging conditions across patients. Images were analyzed using the dedicated software KINOSIS (Konica Minolta, Japan), and among the automatically extracted parameters were the following: LV max area (maximum inspiratory lung area; largest single‑frame value across the entire DDR sequence, cm^2^), LV min area (minimum expiratory lung area; smallest single‑frame value across the entire sequence, cm^2^), and respiratory rate (breaths per minute). LV max and LV min were extracted independently and did not necessarily originate from the same respiratory cycle.

### Extubation readiness assessment

Extubation readiness was determined by the treating intensivists based on a comprehensive assessment of each patient’s clinical condition. In general, this assessment included hemodynamic stability, adequate oxygenation and ventilation with reduced ventilatory support, an appropriate level of consciousness for airway protection, manageable airway secretions, and sufficient cough strength. A mandatory standardized weaning protocol, such as a uniform T-piece trial, was not applied to all patients.

### Outcomes and variables

The primary outcome was post-extubation respiratory deterioration, defined as any increase in oxygen supplementation, including an increase in FIO₂, before ICU discharge. As a supportive analysis, we defined a secondary respiratory support/oxygenation outcome as post-extubation NPPV or NHF use or post-extubation PaO₂/FIO₂ ≤ 300.

The following DDR-derived variables were evaluated: pre-extubation maximum lung area, pre-extubation minimum lung area, pre-extubation lung-area excursion, post-extubation maximum lung area, post-extubation minimum lung area, post-extubation lung-area excursion, and the change in lung-area excursion from before to after extubation. Lung-area excursion was calculated as maximum lung area minus minimum lung area.

The following baseline clinical variables were extracted from electronic medical records and ICU charts: age, sex, height, weight, body mass index, SOFA score, diabetes mellitus, hypertension, emergency admission status, and admission department. Detailed respiratory and cardiopulmonary comorbidities were also extracted, including chronic obstructive pulmonary disease, heart failure, interstitial pneumonia or interstitial lung disease, post-lung resection status, bronchial asthma, postoperative phrenic nerve palsy, aspiration pneumonia, and other respiratory conditions.

Respiratory support and oxygenation variables included PaO₂/FIO₂ before and after extubation, post-extubation NPPV or NHF use, reintubation before ICU discharge, and days from extubation to ICU discharge. Ventilatory and weaning-related variables at the time of pre-extubation assessment or DDR imaging included days from ICU admission to extubation, ventilation mode, FIO₂, positive end-expiratory pressure, pressure support, ventilator-chart respiratory rate, and tidal volume.

### Statistical analysis

Continuous variables (median [interquartile range]) and categorical variables (counts, %) were compared between patients with and without post-extubation respiratory deterioration using the Mann–Whitney U test and Fisher’s exact test, respectively. To evaluate the association between DDR-related variables and the primary outcome, univariate and multivariable logistic regression analyses were conducted. Covariates in these models were selected from clinical perspective. To confirm the robustness of the models, several covariate patterns were examined. Due to the small sample size, all logistic regression models were corrected using Firth’s penalized likelihood method to address potential estimation bias and quasi-complete separation [10].

Furthermore, to explore the performance of DDR-derived features and clinical variables on primary outcome prediction, we applied XGBoost (eXtreme Gradient Boosting), a tree-based ensemble learning method that captures nonlinear relationships and interactions between variables while suppressing overfitting using depth limitation, L1/L2 regularization, and row/column subsampling [11]. Feature importance—quantified by the xgb.importance function included in the xgboost R package (v1.7.7)—and SHapley Additive exPlanations (SHAP) [12] were calculated to visualize the contribution of each variable to the model output. The XGBoost/SHAP analysis was conducted as an exploratory, hypothesis-generating analysis to examine the relative contribution of candidate variables within a prediction model. Given the limited sample size and small number of outcome events, this analysis was not intended to establish a validated prediction model or to provide confirmatory evidence of independent associations. SHAP values were interpreted as model-based feature contributions rather than measures of statistical significance.

All statistical analyses were performed using R version 4.4.2 (R Foundation for Statistical Computing, Vienna, Austria). In all analyses, statistical significance was defined as p < 0.05. Given the exploratory nature of this study, no adjustments for multiple comparisons were applied.

## Results

A total of 56 patients were included in the analysis. The median age was 71 years [IQR, 63–80], and 59% were male. Respiratory deterioration was observed in 10 patients (18%), and reintubation was required in only 1 patient (2%). Most patients were admitted under surgical departments, with 52 of 56 patients (92.9%) classified as surgical admissions. Detailed respiratory and cardiopulmonary comorbidities are shown in Table 1; no patients had interstitial pneumonia or interstitial lung disease. Ventilatory and weaning-related conditions before extubation are summarized in S1 Table. Before extubation or DDR imaging, 42 of 56 patients (75.0%) were managed with PSV/CPAP, and no patients underwent a T-piece trial.

**Table 1:** Baseline characteristics and DDR-derived indices stratified by post-extubation respiratory deterioration.

Variable	No Deterioration	Deterioration	*p*
	(N = 46)	(N = 10)	
Age, yr	68.5 [61.5, 76.5]	80.0 [73.0, 84.2]	0.005
Sex, male	28 (60.9%)	5 (50.0%)	0.725
Height, cm	165.0 [153.5, 169.6]	157.5 [148.5, 168.1]	0.335
Weight, kg	58.5 [49.2, 73.7]	56.0 [45.8, 59.1]	0.266
Body mass index, kg/m²	22.6 [20.5, 26.4]	21.2 [20.4, 22.2]	0.271
SOFA score	12.0 [10.2, 13.0]	14.0 [12.2, 15.5]	0.014
Diabetes mellitus	14 (30.4%)	1 (10.0%)	0.259
Hypertension	21 (45.7%)	3 (30.0%)	0.489
Any listed cardiopulmonary/respiratory condition	13 (28.3%)	2 (20.0%)	0.713
COPD	2 (4.3%)	0 (0.0%)	>0.999
Heart failure	3 (6.5%)	1 (10.0%)	0.556
Interstitial pneumonia / interstitial lung disease	0 (0.0%)	0 (0.0%)	—
Post-lung resection status	1 (2.2%)	0 (0.0%)	>0.999
Bronchial asthma	2 (4.3%)	0 (0.0%)	>0.999
Postoperative phrenic nerve palsy	1 (2.2%)	0 (0.0%)	>0.999
Aspiration pneumonia	2 (4.3%)	1 (10.0%)	0.452
Other respiratory condition	3 (6.5%)	0 (0.0%)	>0.999
Emergency admission	25 (54.3%)	9 (90.0%)	0.070
Department			>0.999
Internal	2 (4.3%)	0 (0.0%)	
Surgical	42 (91.3%)	10 (100.0%)	
Other	2 (4.3%)	0 (0.0%)	
Respiratory rate, /min (pre)	15.0 [12.0, 21.0]	21.0 [18.0, 26.2]	0.039
Respiratory rate, /min (post)	21.0 [15.0, 27.0]	22.5 [18.0, 35.2]	0.338
Maximum lung area, cm² (pre)	312.8 [270.3, 368.4]	324.0 [263.3, 338.5]	0.839
Minimum lung area, cm² (pre)	276.0 [217.4, 328.1]	264.9 [232.7, 298.7]	>0.999
Pre-extubation lung-area excursion, cm²	40.7 [28.2, 55.2]	38.7 [33.5, 46.0]	0.692
Maximum lung area, cm² (post)	305.5 [250.2, 354.8]	291.2 [251.1, 315.9]	0.330
Minimum lung area, cm² (post)	264.3 [212.7, 300.1]	264.7 [217.3, 283.6]	0.630
Post-extubation lung-area excursion, cm²	38.3 [32.3, 47.1]	35.3 [27.1, 38.7]	0.111
Change in lung-area excursion, cm² (Post–Pre)	0.9 [−15.4, 9.6]	−3.8 [−12.6, 3.7]	0.330

Values are median [IQR] or n (%). P values were calculated using the Mann–Whitney U test for continuous variables and Fisher’s exact test for categorical variables. Lung-area excursion was calculated as maximum lung area minus minimum lung area extracted from DDR images. SOFA, Sequential Organ Failure Assessment; DDR, dynamic digital radiography.

Compared to the non-deterioration group, the deterioration group was significantly older (80.0 [73.0–84.2] vs. 68.5 [61.5–76.5] years, p = 0.005) and had a higher pre-extubation respiratory rate (21 [18–26] vs. 15 [12–21] breaths/min, p = 0.038). In contrast, no significant intergroup differences were observed in other direct DDR-derived parameters such as LV max area or LV min area, either before or after extubation (Table 1).

Objective oxygenation and respiratory support outcomes are summarized‌‌ in Table 2. The deterioration group had a lower post-extubation PaO₂/FIO₂ ratio than the non-deterioration group (272.0 [237.3–325.8] vs. 348.5 [296.7–451.1], p = 0.024), and post-extubation PaO₂/FIO₂ ≤ 300 was more frequent in the deterioration group (70.0% vs. 29.5%, p = 0.028). Post-extubation NPPV or NHF use was also more frequent in the deterioration group (90.0% vs. 8.7%, p < 0.001). Reintubation before ICU discharge occurred in only one patient.

**Table 2:** Objective oxygenation indices and reintubation according to post-extubation respiratory deterioration.

Variable	No deterioration	Deterioration	*p*
	(n = 46)	(n = 10)	
PaO₂/FIO₂ before extubation	375.7 [286.8, 439.8]	290.5 [251.4, 346.1]	0.054
PaO₂/FIO₂ ≤ 300 before extubation	13 (28.3%)	6 (60.0%)	0.073
PaO₂/FIO₂ after extubation	348.5 [296.7, 451.1]	272.0 [237.3, 325.8]	0.024
PaO₂/FIO₂ ≤ 300 after extubation	13 (29.5%)	7 (70.0%)	0.028
Change in PaO₂/FIO₂ (post − pre)	−22.5 [−55.5, 61.0]	−6.0 [−14.3, 3.7]	0.555
Reintubation before ICU discharge	0 (0.0%)	1 (10.0%)	0.179
Post-extubation NPPV or NHF use	4 (8.7%)	9 (90.0%)	<0.001
Days from extubation to ICU discharge	2.0 [1.0, 3.0]	5.0 [4.0, 5.0]	<0.001

Values are median [IQR] or n (%). P values were calculated using the Mann–Whitney U test for continuous variables and Fisher’s exact test for categorical variables. Days from extubation to ICU discharge was calculated as ICU discharge date minus extubation date; for the single reintubated patient, the value was coded as 5 days according to the original analysis definition and should not be interpreted as conventional 28-day ventilator-free days. Post-extubation NPPV or NHF use refers to respiratory support use before ICU discharge. ICU, intensive care unit; NPPV, noninvasive positive-pressure ventilation; NHF, nasal high-flow oxygen therapy.

### Multivariable logistic regression analysis

In univariate logistic regression, the odds ratio for post-extubation lung-area excursion was 0.95 per 1 cm^2^ increase (95% CI, 0.89–1.00; p = 0.080; Table 3). After adjustment for age, the association was attenuated and was not statistically significant, with an odds ratio of 0.98 per 1 cm^2^ increase (95% CI, 0.92–1.03; p = 0.431). The confidence interval crossed 1, indicating that this result should be interpreted cautiously as exploratory rather than confirmatory evidence of an independent association. In additional models adjusted individually for sex, post-extubation respiratory rate, height, or emergency admission, the estimates were generally similar, but none demonstrated a statistically significant independent association between post-extubation lung-area excursion and respiratory deterioration. When the secondary respiratory support/oxygenation outcome was used, the regression-based estimates for post-extubation lung-area excursion were generally in the same direction as those for the primary outcome, but they did not reach statistical significance (S2 Table). Additional exploratory sensitivity analyses adjusting for severity, baseline oxygenation, comorbidities, and ventilatory or weaning-related variables showed generally similar directions of association, although these analyses were limited by the small number of outcome events (S3 Table).

**Table 3:** Association between post-extubation lung-area excursion and respiratory deterioration: Univariate and multivariable logistic regression.

	Univariate Analysis	Adjusted OR [95% CI] (p value) in Multivariable Analysis				
Variable	OR [95% CI](p value)	Model 1	Model 2	Model 3	Model 4	Model 5
Post-Extubation Lung-Area Excursion, cm²	0.95 [0.89–1.00](0.080)	0.98 [0.92–1.03](0.431)	0.95 [0.88–1.01](0.094)	0.95 [0.88–1.00](0.064)	0.95 [0.87–1.01](0.132)	0.96 [0.89–1.01](0.102)
Age, yr	1.11 [1.03–1.22](0.005)	1.09 [1.01–1.21](0.021)	--	--	--	--
Sex, Male (1/0)	0.65 [0.17–2.49](0.521)	--	1.31 [0.28–6.53](0.733)	--	--	--
Respiratory Rate, breaths/min (post)	1.03 [0.97–1.10](0.302)	--	--	1.05 [0.97–1.13](0.205)	--	--
Height, cm	0.97 [0.91–1.03](0.358)	--	--	--	1.01 [0.93–1.09](0.837)	--
Emergency Admission	5.34 [1.09–52.65](0.038)	--	--	--	--	5.18 [1.03–51.46](0.045)

The univariate model includes a single predictor. For multivariable models, covariates were added individually as follows: Model 1, age; Model 2, sex; Model 3, post-extubation respiratory rate; Model 4, height; Model 5, emergency admission.

### Exploratory predictive analysis using machine learning: XGBoost and SHAP

**Fig 2:**
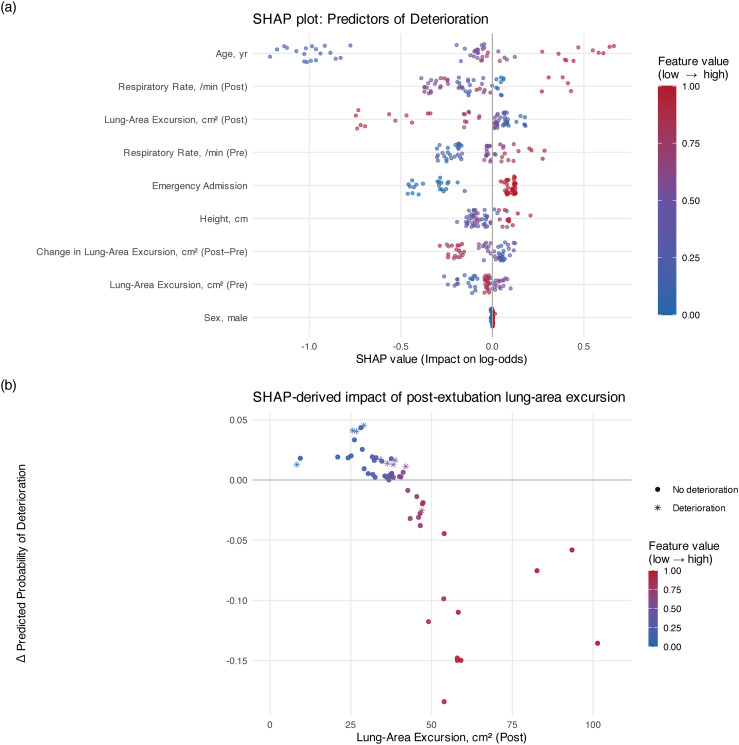
Exploratory SHAP-based interpretation of the XGBoost model for post-extubation respiratory deterioration. (a) SHAP summary plot from the exploratory XGBoost model trained on clinical and DDR-derived variables. The x-axis shows SHAP values, indicating each feature’s model-based contribution to the predicted log-odds of respiratory deterioration. Dot color reflects the feature value, with blue indicating lower values and red indicating higher values. **(b)** SHAP-derived impact of post-extubation lung-area excursion on the predicted probability of respiratory deterioration. Each point represents one patient. These SHAP-based results indicate feature contributions within an exploratory prediction model and should not be interpreted as evidence of statistical significance or independent association.

We further explored model-based feature contributions using XGBoost and SHAP. In the SHAP summary plot, age, post-extubation respiratory rate, and post-extubation lung-area excursion showed relatively large model-based contributions within the exploratory prediction model (Fig 2a). The SHAP-derived impact plot suggested that lower post-extubation lung-area excursion values tended to contribute to model predictions in favor of respiratory deterioration, whereas higher values tended to contribute in the opposite direction (Fig 2b). These findings should be interpreted as feature contributions within an exploratory prediction model and not as evidence of independent statistical association or statistical significance. In the exploratory XGBoost/SHAP analysis for the secondary respiratory support/oxygenation outcome, post-extubation lung-area excursion also showed a relatively large model-based contribution (S1 Fig). This finding was interpreted as exploratory evidence of consistency under an alternative outcome definition, not as confirmatory validation.

## Discussion

In this prospectively collected, single-center cohort of 56 consecutive adult ICU extubation cases, we examined associations between DDR-derived parameters around extubation and post-extubation respiratory deterioration. Directly measured DDR-derived parameters did not differ significantly between patients with and without respiratory deterioration. In logistic regression analysis, post-extubation lung-area excursion was not statistically significant after adjustment for age. In exploratory XGBoost/SHAP analysis, age, post-extubation respiratory rate, and post-extubation lung-area excursion showed relatively large model-based contributions. These findings suggest that DDR-derived dynamic information may contain exploratory predictive signal, but they do not establish post-extubation lung-area excursion as an independent or validated predictor.

An important point in interpreting the present findings is the distinction between regression-based association and model-based feature contribution. Logistic regression was used to estimate the association between post-extubation lung-area excursion and respiratory deterioration while accounting for individual covariates, including age. In this analysis, the DDR-derived variable was not statistically significant after adjustment for age. In contrast, the XGBoost/SHAP analysis evaluated how each variable contributed to predictions within an exploratory multivariable machine-learning model. Therefore, the relatively high SHAP ranking of post-extubation lung-area excursion should not be interpreted as evidence of statistical significance or as confirmation of an independent association.

The supportive analysis using a secondary respiratory support/oxygenation outcome showed broadly similar directional findings, and post-extubation lung-area excursion also showed a relatively large model-based contribution in the exploratory SHAP analysis. However, because this analysis was performed in the same small cohort and without external validation, it should be regarded as a consistency check under an alternative outcome definition rather than validation of the DDR-derived marker.

Low post-extubation lung-area excursion values may reflect reduced pulmonary ventilation capacity and/or weakened respiratory effort. This diminished “breathing amplitude” may also indicate reduced respiratory reserve. Such a state may result from impaired diaphragmatic function, decreased thoracic compliance, limited spontaneous breathing effort, or impaired central respiratory drive [13]. In the immediate post-extubation period, patients must transition from assisted to spontaneous breathing, and reduced lung excursion on DDR may reflect insufficient ventilatory reserve during this transition. In particular, elderly patients or those with prolonged intubation are prone to diaphragmatic fatigue, muscle atrophy, pain, or altered consciousness, all of which can limit spontaneous ventilation [13].

In our study, age and pre-extubation respiratory rate differed significantly between patients with and without respiratory deterioration, consistent with previous reports. Older patients often exhibit reduced respiratory muscle strength, lower pulmonary reserve, sarcopenia, and increased aspiration risk [2,14]. An elevated respiratory rate before extubation may reflect increased respiratory effort or fatigue and is considered a sign of impending extubation failure [14,15]. These simple clinical variables remain important for post-extubation risk assessment and should be considered alongside, rather than replaced by, DDR-derived dynamic information.

Traditionally, extubation decisions and risk assessments have relied on respiratory physiological indices such as the RSBI, maximal inspiratory pressure, and vital capacity [4]. However, these tests require patient cooperation and may be technically burdensome. Although lung ultrasound has gained attention in recent years, its limitations include operator dependence, variable reproducibility, and difficulty in obtaining interpretable images in some patients [16]. In contrast, DDR offers a low-dose, non-invasive modality capable of visualizing pulmonary dynamics quantitatively and intuitively at the bedside. DDR should not be regarded as a replacement for simple clinical variables such as age or respiratory rate. Rather, DDR may add functional information that is difficult to capture using single-point clinical variables alone, including dynamic changes in ventilation, laterality differences, and coordination of respiratory motion before and after extubation.

Radiation exposure and institutional cost are also important considerations. DDR is a low-dose radiographic technique, but its clinical use should be justified by the additional functional information it provides. A recent state-of-the-art review of dynamic chest radiography similarly emphasized that this modality provides low-dose dynamic imaging information on thoracic motion, ventilation, and perfusion, while also noting that further research is needed to define its clinical niche [17]. At our institution, DDR can be performed using the existing radiographic workflow without additional imaging costs beyond standard chest radiography; however, the clinical and practical value of DDR requires further evaluation in larger studies.

In the present study, we focused on lung-area excursion because it can be automatically derived from DDR and provides a simple quantitative representation of respiratory-related changes in lung area. This parameter may be practical for repeated bedside assessment in ICU patients because it requires minimal complex post-processing and is less dependent on subjective interpretation. Previous studies using other imaging modalities have suggested that extubation failure is associated with reduced lung aeration or a smaller ventilated lung region. For example, Joussellin et al. reported that patients with extubation failure had higher lung ultrasound scores and lower surface available for ventilation measured by electrical impedance tomography [18], and Dimitriou et al. reported that lung area on post-extubation chest radiographs was associated with extubation outcomes in neonates [19]. However, DDR-derived lung-area excursion itself has not been sufficiently validated as a predictor of post-extubation respiratory deterioration. Therefore, the present study should be interpreted as an exploratory investigation of whether dynamic changes in lung area around extubation may be clinically informative.

### Limitations

This study has several limitations. The most important methodological limitation is the limited sample size, particularly the small number of respiratory deterioration events. Because the XGBoost/SHAP analysis was applied to 56 patients with only 10 outcome events, the risk of overfitting is substantial. Although this analysis was useful for exploratory assessment of model-based feature contribution, the resulting feature rankings may be unstable and require validation in larger, independent, preferably multicenter cohorts. Therefore, the machine-learning findings should be interpreted as hypothesis-generating and should not be used as evidence of a validated prediction model. In addition, although data were prospectively collected in a consecutive, single-center cohort, the influence of selection bias or residual confounding cannot be completely excluded. Because most patients were admitted under surgical departments, the findings may not be directly generalizable to medical ICU populations, in which indications for intubation, duration of mechanical ventilation, and mechanisms of extubation failure may differ. Indication for intubation, prior intubation history, steroid exposure, and long-term sedation were not sufficiently standardized for inclusion in the main analysis and may represent additional sources of residual confounding.

The primary outcome, defined as an increase in oxygen supplementation before ICU discharge, includes an element of clinical judgment and may not fully capture objective physiological deterioration. To address this limitation, we added objective oxygenation and respiratory support outcomes, including post-extubation PaO₂/FIO₂, post-extubation PaO₂/FIO₂ ≤ 300, post-extubation NPPV or NHF use, and reintubation before ICU discharge. However, these analyses were also exploratory, and reintubation could not be meaningfully analyzed because only one patient required reintubation before ICU discharge.

All DDR examinations were performed in the supine position to standardize imaging conditions; however, this position may have influenced respiratory mechanics through effects on diaphragmatic motion and intra-abdominal pressure.

## Conclusions

In this exploratory ICU cohort, post-extubation lung-area excursion derived from DDR was not statistically significant after adjustment for age, but it showed a relatively large model-based contribution in exploratory XGBoost/SHAP analysis. These findings suggest that DDR-derived dynamic respiratory information may provide complementary, hypothesis-generating information for post-extubation assessment. Larger prospective studies with external validation are required before DDR-derived indices can be considered clinically validated predictors.

## Supporting information

S1 File Supplemental materials. (PDF)

S1 Table Ventilatory and weaning-related conditions before extubation. (PDF)

S2 Table Comparison of the association of post-extubation lung-area excursion with the primary outcome and a secondary respiratory support/oxygenation outcome. (PDF)

S3 Table Sensitivity analyses of the association between post-extubation lung-area excursion and post-extubation respiratory deterioration after additional covariate adjustment. (PDF)

S1 Fig SHAP summary plot for the secondary respiratory support/oxygenation outcome. SHAP summary plot from the XGBoost model trained to predict the secondary respiratory support/oxygenation outcome, defined as NPPV/NHF use or post-extubation PaO₂/FIO₂ ≤ 300. The model used the same predictor set and hyperparameter structure as the primary XGBoost analysis, with the outcome replaced by the secondary outcome. Each point represents one patient. The x-axis shows the SHAP value, indicating each feature’s impact on the model output on the log-odds scale. Dot color reflects the feature value, with blue indicating lower values and red indicating higher values. Variables are ordered according to Gain-based feature importance in this secondary-outcome model. NPPV, noninvasive positive-pressure ventilation; NHF, nasal high-flow oxygen therapy; SHAP, Shapley additive explanations; PaO₂/FIO₂, ratio of arterial oxygen partial pressure to fractional inspired oxygen.
(PDF)
