# Supplementary material for: Association between dynamic digital radiography findings and post-extubation respiratory deterioration: A retrospective exploratory analysis of a prospectively collected ICU cohort
Source: PLoS One. 2026 Jun 22;21(6):e0352029. doi: 10.1371/journal.pone.0352029 (PMC13286171; doi:10.1371/journal.pone.0352029)

Supplemental Materials

Association between dynamic digital radiography findings and post-extubation respiratory deterioration: A retrospective exploratory analysis of a prospectively collected ICU cohort

Satoshi Komatsu, Kazuki Nishida, Yoshitaka Hara, Naohide Kuriyama, and Tomoyuki Nakamura

This supplemental file provides additional analyses supporting the interpretation and robustness of the findings presented in the main manuscript. The materials include peri-extubation ventilatory and weaning-related conditions, analyses using a secondary respiratory support/oxygenation outcome, additional covariate-adjusted sensitivity analyses, and exploratory SHAP-based model interpretation for the secondary outcome. These analyses provide further clinical context for the study population and assess whether the findings are consistent under alternative but clinically relevant outcome and adjustment settings.

Contents

Table S1. Ventilatory and weaning-related conditions before extubation.
Table S2. Comparison of the association of post-extubation lung-area excursion with the primary outcome and a secondary respiratory support/oxygenation outcome.
Table S3. Sensitivity analyses of the association between post-extubation lung-area excursion and post-extubation respiratory deterioration after additional covariate adjustment.
Figure S1. SHAP summary plot for the secondary respiratory support/oxygenation outcome.

Definitions and abbreviations

The secondary respiratory support/oxygenation outcome was defined as NPPV/NHF use or post-extubation $PaO_2/FIO_2 \leq 300$.

DDR, dynamic digital radiography; NPPV, noninvasive positive-pressure ventilation; NHF, nasal high-flow oxygen therapy; SHAP, Shapley additive explanations; PaO_2/FIO_2, ratio of arterial oxygen partial pressure to fractional inspired oxygen.

Table S1. Ventilatory and weaning-related conditions before extubation.

Variable	No deterioration (N = 46)	Deterioration (N = 10)	P
Days from ICU admission to extubation, d	1.0 [1.0, 2.0]	1.0 [1.0, 2.8]	>0.999
Ventilation mode before extubation/DDR (%)			0.698
PSV/CPAP	35 (76.1)	7 (70.0)	
T-piece	0 (0.0)	0 (0.0)	
Assisted/controlled	11 (23.9)	3 (30.0)	
Other/unclear	0 (0.0)	0 (0.0)	
FIO2 before extubation, fraction	0.30 [0.25, 0.40]	0.35 [0.30, 0.40]	0.319
PEEP before extubation, cmH2O	5.0 [5.0, 5.8]	5.0 [5.0, 7.8]	0.313
Pressure support before extubation, cmH2O	5.5 [5.0, 10.0]	9.0 [8.0, 10.0]	0.111
Ventilator-chart respiratory rate before extubation, /min	17.5 [14.0, 21.8]	19.5 [15.0, 22.8]	0.653
Tidal volume before extubation, mL	439.5 [347.0, 502.2]	402.5 [358.5, 515.0]	0.974

Values are presented as median [IQR] or n (%). Continuous variables were compared using the Mann–Whitney U test; categorical variables were compared using Fisher's exact test. Ventilation modes were grouped from the recorded pre-extubation ventilator model. PSV/CPAP includes pressure support ventilation or CPAP-like spontaneous breathing modes. The intubated-at-ICU-admission variable was omitted from the table if all values were missing. DDR, dynamic digital radiography; PSV, pressure support ventilation; CPAP, continuous positive airway pressure; PEEP, positive end-expiratory pressure.

Table S2. Comparison of the association of post-extubation lung-area excursion with the primary outcome and a secondary respiratory support/oxygenation outcome.

Model	Adjustment	**Primary outcome** OR [95% CI] (P-value)	**Secondary outcome** OR [95% CI] (P-value)
Unadjusted	None	0.95 [0.89–1.00] (0.080)	0.97 [0.93–1.01] (0.119)
Model 1	Age	0.98 [0.92–1.03] (0.431)	0.98 [0.94–1.01] (0.199)
Model 2	Sex (male)	0.95 [0.88–1.01] (0.094)	0.97 [0.93–1.01] (0.151)
Model 3	Post-extubation respiratory rate	0.95 [0.88–1.00] (0.064)	0.97 [0.92–1.00] (0.092)
Model 4	Height	0.95 [0.87–1.01] (0.132)	0.96 [0.91–1.00] (0.061)
Model 5	Emergency admission	0.96 [0.89–1.01] (0.102)	0.97 [0.93–1.01] (0.127)

Primary outcome: respiratory deterioration.

Secondary outcome: NPPV/NHF or post-extubation $PaO_2/FIO_2 \leq 300$.

OR, odds ratio; CI, confidence interval; NPPV, noninvasive positive pressure ventilation; NHF, nasal high flow.

Table S3. Sensitivity analyses of the association between post-extubation lung-area excursion and post-extubation respiratory deterioration after additional covariate adjustment.

Domain	Adjustment	OR for post-extubation lung-area excursion, per 1 cm² [95% CI] (P value)
Unadjusted	None	0.95 [0.89–1.00] (0.080)
Original Table 2 covariates	Age, yr	0.98 [0.92–1.03] (0.431)
	Sex, male	0.95 [0.88–1.01] (0.094)
	Respiratory rate, /min (post)	0.95 [0.88–1.00] (0.064)
	Height, cm	0.95 [0.87–1.01] (0.132)
	Emergency admission	0.96 [0.89–1.01] (0.102)
Severity / baseline status	SOFA score	0.94 [0.87–1.00] (0.065)
	PaO2/FIO2 before extubation	0.96 [0.90–1.01] (0.142)
	Weight, kg	0.96 [0.89–1.02] (0.188)
	Body-mass index, kg/m²	0.96 [0.89–1.01] (0.117)
	Surgical department	0.96 [0.90–1.01] (0.143)
Comorbidity	Diabetes mellitus	0.95 [0.89–1.01] (0.089)
	Hypertension	0.96 [0.89–1.01] (0.114)
	Any listed cardiopulmonary or respiratory condition	0.95 [0.89–1.01] (0.093)
	Any listed respiratory condition	0.96 [0.89–1.01] (0.093)
	COPD	0.95 [0.89–1.01] (0.087)
	Heart failure	0.95 [0.89–1.00] (0.074)
	Interstitial pneumonia/lung disease	Not estimable
	Post-lung resection status	0.95 [0.89–1.00] (0.078)
	Bronchial asthma	0.95 [0.89–1.01] (0.089)
	Postoperative phrenic nerve palsy	0.95 [0.89–1.00] (0.081)
	Aspiration pneumonia	0.95 [0.89–1.01] (0.087)
	Other respiratory condition	0.96 [0.89–1.01] (0.109)
Ventilation / weaning	Days from ICU admission to extubation	0.95 [0.89–1.01] (0.091)
	Assisted/controlled mode before extubation	0.96 [0.89–1.01] (0.090)
	FIO2 before extubation, fraction	0.95 [0.89–1.00] (0.081)

PEEP before extubation, cmH2O	0.94 [0.87–1.00] (0.032)
Pressure support before extubation, cmH2O	0.96 [0.90–1.01] (0.112)
Ventilator-chart respiratory rate before extubation	0.95 [0.89–1.01] (0.092)
Tidal volume before extubation, mL	0.95 [0.88–1.00] (0.076)
Respiratory rate, /min (pre)	0.95 [0.89–1.00] (0.068)

Values are odds ratios for post-extubation lung-area excursion per 1 cm^2 increase. Each model included post-extubation lung-area excursion and one additional covariate listed in the Adjustment column. Firth-corrected logistic regression was used because of the small number of outcome events. “Not estimable” indicates that the model could not be estimated because the added covariate had no variation or insufficient information.

Figure S1. SHAP summary plot for the secondary respiratory support/oxygenation outcome.

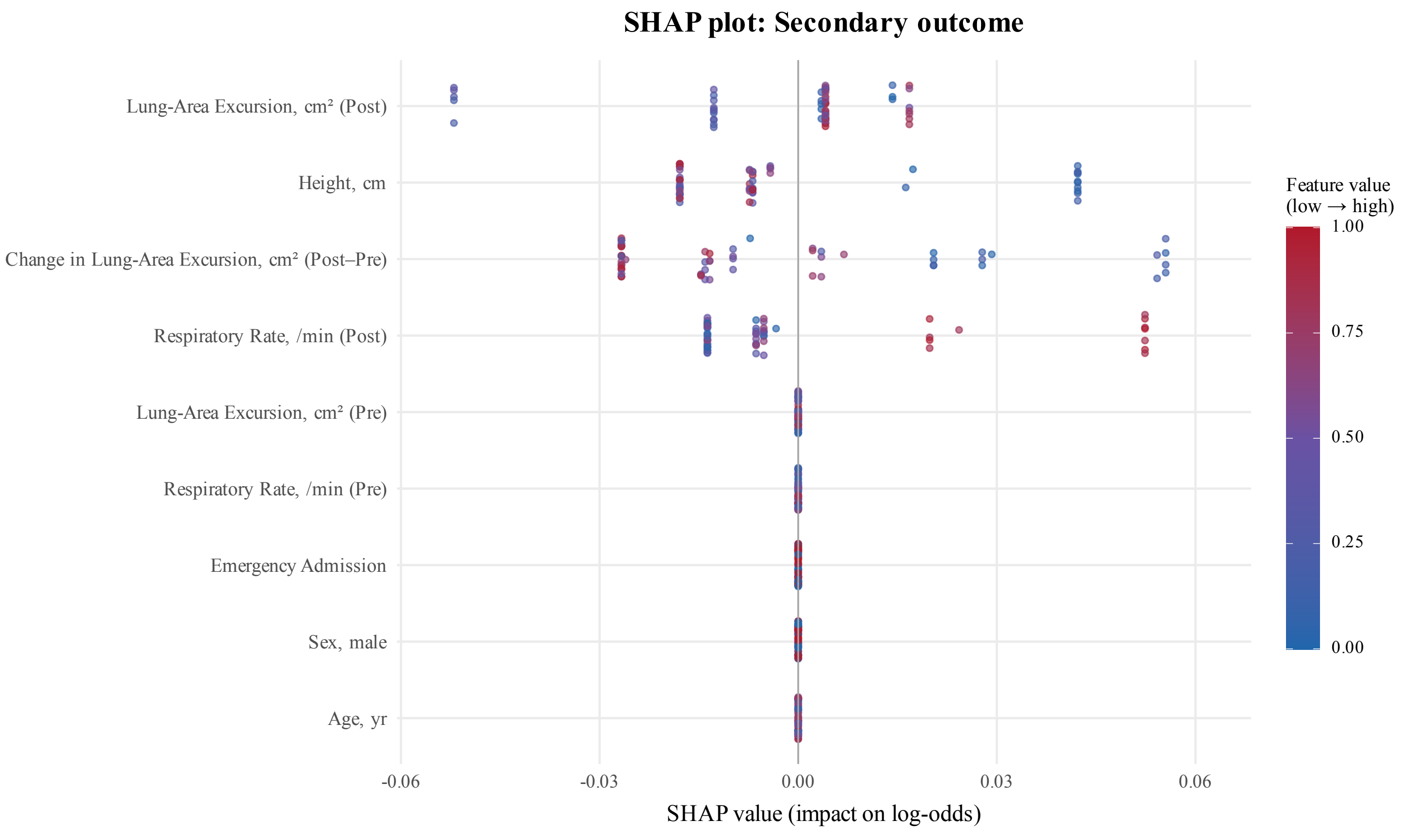

Supplement: S1 File — (PDF) [file pone.0352029.s001.pdf]
