# Supplementary material for: Association between dynamic digital radiography findings and post-extubation respiratory deterioration: A retrospective exploratory analysis of a prospectively collected ICU cohort
Source: PLoS One. 2026 Jun 22;21(6):e0352029. doi: 10.1371/journal.pone.0352029 (PMC13286171; doi:10.1371/journal.pone.0352029)
Supplement: S1 Table — (PDF) [file pone.0352029.s002.pdf]

Table S1. Ventilatory and weaning-related conditions before extubation.

Variable	No deterioration (N = 46)	Deterioration (N = 10)	P
Days from ICU admission to extubation, d	1.0 [1.0, 2.0]	1.0 [1.0, 2.8]	>0.999
Ventilation mode before extubation/DDR (%)			0.698
PSV/CPAP	35 (76.1)	7 (70.0)	
T-piece	0 (0.0)	0 (0.0)	
Assisted/controlled	11 (23.9)	3 (30.0)	
Other/unclear	0 (0.0)	0 (0.0)	
FIO2 before extubation, fraction	0.30 [0.25, 0.40]	0.35 [0.30, 0.40]	0.319
PEEP before extubation, cmH2O	5.0 [5.0, 5.8]	5.0 [5.0, 7.8]	0.313
Pressure support before extubation, cmH2O	5.5 [5.0, 10.0]	9.0 [8.0, 10.0]	0.111
Ventilator-chart respiratory rate before extubation, /min	17.5 [14.0, 21.8]	19.5 [15.0, 22.8]	0.653
Tidal volume before extubation, mL	439.5 [347.0, 502.2]	402.5 [358.5, 515.0]	0.974

Values are presented as median [IQR] or n (%). Continuous variables were compared using the Mann–Whitney U test; categorical variables were compared using Fisher's exact test. Ventilation modes were grouped from the recorded pre-extubation ventilator model. PSV/CPAP includes pressure support ventilation or CPAP-like spontaneous breathing modes. The intubated-at-ICU-admission variable was omitted from the table if all values were missing. DDR, dynamic digital radiography; PSV, pressure support ventilation; CPAP, continuous positive airway pressure; PEEP, positive end-expiratory pressure.
