# Supplementary material for: Association between dynamic digital radiography findings and post-extubation respiratory deterioration: A retrospective exploratory analysis of a prospectively collected ICU cohort
Source: PLoS One. 2026 Jun 22;21(6):e0352029. doi: 10.1371/journal.pone.0352029 (PMC13286171; doi:10.1371/journal.pone.0352029)
Supplement: S2 Table — (PDF) [file pone.0352029.s003.pdf]

Table S2. Comparison of the association of post-extubation lung-area excursion with the primary outcome and a secondary respiratory support/oxygenation outcome.

Model	Adjustment	**Primary outcome** OR [95% CI] (P-value)	**Secondary outcome** OR [95% CI] (P-value)
Unadjusted	None	0.95 [0.89–1.00] (0.080)	0.97 [0.93–1.01] (0.119)
Model 1	Age	0.98 [0.92–1.03] (0.431)	0.98 [0.94–1.01] (0.199)
Model 2	Sex (male)	0.95 [0.88–1.01] (0.094)	0.97 [0.93–1.01] (0.151)
Model 3	Post-extubation respiratory rate	0.95 [0.88–1.00] (0.064)	0.97 [0.92–1.00] (0.092)
Model 4	Height	0.95 [0.87–1.01] (0.132)	0.96 [0.91–1.00] (0.061)
Model 5	Emergency admission	0.96 [0.89–1.01] (0.102)	0.97 [0.93–1.01] (0.127)

Primary outcome: respiratory deterioration.

Secondary outcome: NPPV/NHF or post-extubation $PaO_2/FIO_2 \leq 300$.

OR, odds ratio; CI, confidence interval; NPPV, noninvasive positive pressure ventilation; NHF, nasal high flow.
