# Supplementary material for: Association between dynamic digital radiography findings and post-extubation respiratory deterioration: A retrospective exploratory analysis of a prospectively collected ICU cohort
Source: PLoS One. 2026 Jun 22;21(6):e0352029. doi: 10.1371/journal.pone.0352029 (PMC13286171; doi:10.1371/journal.pone.0352029)
Supplement: S3 Table — (PDF) [file pone.0352029.s004.pdf]

Table S3. Sensitivity analyses of the association between post-extubation lung-area excursion and post-extubation respiratory deterioration after additional covariate adjustment.

**Domain**	**Adjustment**	**OR for post-extubation lung-area excursion, per 1 cm² [95% CI] (P value)**
Unadjusted	None	0.95 [0.89–1.00] (0.080)
Original Table 2 covariates	Age, yr	0.98 [0.92–1.03] (0.431)
	Sex, male	0.95 [0.88–1.01] (0.094)
	Respiratory rate, /min (post)	0.95 [0.88–1.00] (0.064)
	Height, cm	0.95 [0.87–1.01] (0.132)
	Emergency admission	0.96 [0.89–1.01] (0.102)
Severity / baseline status	SOFA score	0.94 [0.87–1.00] (0.065)
	PaO2/FIO2 before extubation	0.96 [0.90–1.01] (0.142)
	Weight, kg	0.96 [0.89–1.02] (0.188)
	Body-mass index, kg/m²	0.96 [0.89–1.01] (0.117)
	Surgical department	0.96 [0.90–1.01] (0.143)
Comorbidity	Diabetes mellitus	0.95 [0.89–1.01] (0.089)
	Hypertension	0.96 [0.89–1.01] (0.114)
	Any listed cardiopulmonary or respiratory condition	0.95 [0.89–1.01] (0.093)
	Any listed respiratory condition	0.96 [0.89–1.01] (0.093)
	COPD	0.95 [0.89–1.01] (0.087)
	Heart failure	0.95 [0.89–1.00] (0.074)
	Interstitial pneumonia/lung disease	Not estimable
	Post-lung resection status	0.95 [0.89–1.00] (0.078)
	Bronchial asthma	0.95 [0.89–1.01] (0.089)
	Postoperative phrenic nerve palsy	0.95 [0.89–1.00] (0.081)
	Aspiration pneumonia	0.95 [0.89–1.01] (0.087)
	Other respiratory condition	0.96 [0.89–1.01] (0.109)
Ventilation / weaning	Days from ICU admission to extubation	0.95 [0.89–1.01] (0.091)
	Assisted/controlled mode before extubation	0.96 [0.89–1.01] (0.090)
	FIO2 before extubation, fraction	0.95 [0.89–1.00] (0.081)

PEEP before extubation, cmH2O	0.94 [0.87–1.00] (0.032)
Pressure support before extubation, cmH2O	0.96 [0.90–1.01] (0.112)
Ventilator-chart respiratory rate before extubation	0.95 [0.89–1.01] (0.092)
Tidal volume before extubation, mL	0.95 [0.88–1.00] (0.076)
Respiratory rate, /min (pre)	0.95 [0.89–1.00] (0.068)

Values are odds ratios for post-extubation lung-area excursion per 1 cm^2 increase. Each model included post-extubation lung-area excursion and one additional covariate listed in the Adjustment column. Firth-corrected logistic regression was used because of the small number of outcome events. “Not estimable” indicates that the model could not be estimated because the added covariate had no variation or insufficient information.
