# Supplementary figures and images for: Association between dynamic digital radiography findings and post-extubation respiratory deterioration: A retrospective exploratory analysis of a prospectively collected ICU cohort
Source: PLoS One. 2026 Jun 22;21(6):e0352029. doi: 10.1371/journal.pone.0352029 (PMC13286171; doi:10.1371/journal.pone.0352029)

Figure S1. SHAP summary plot for the secondary respiratory support/oxygenation outcome.

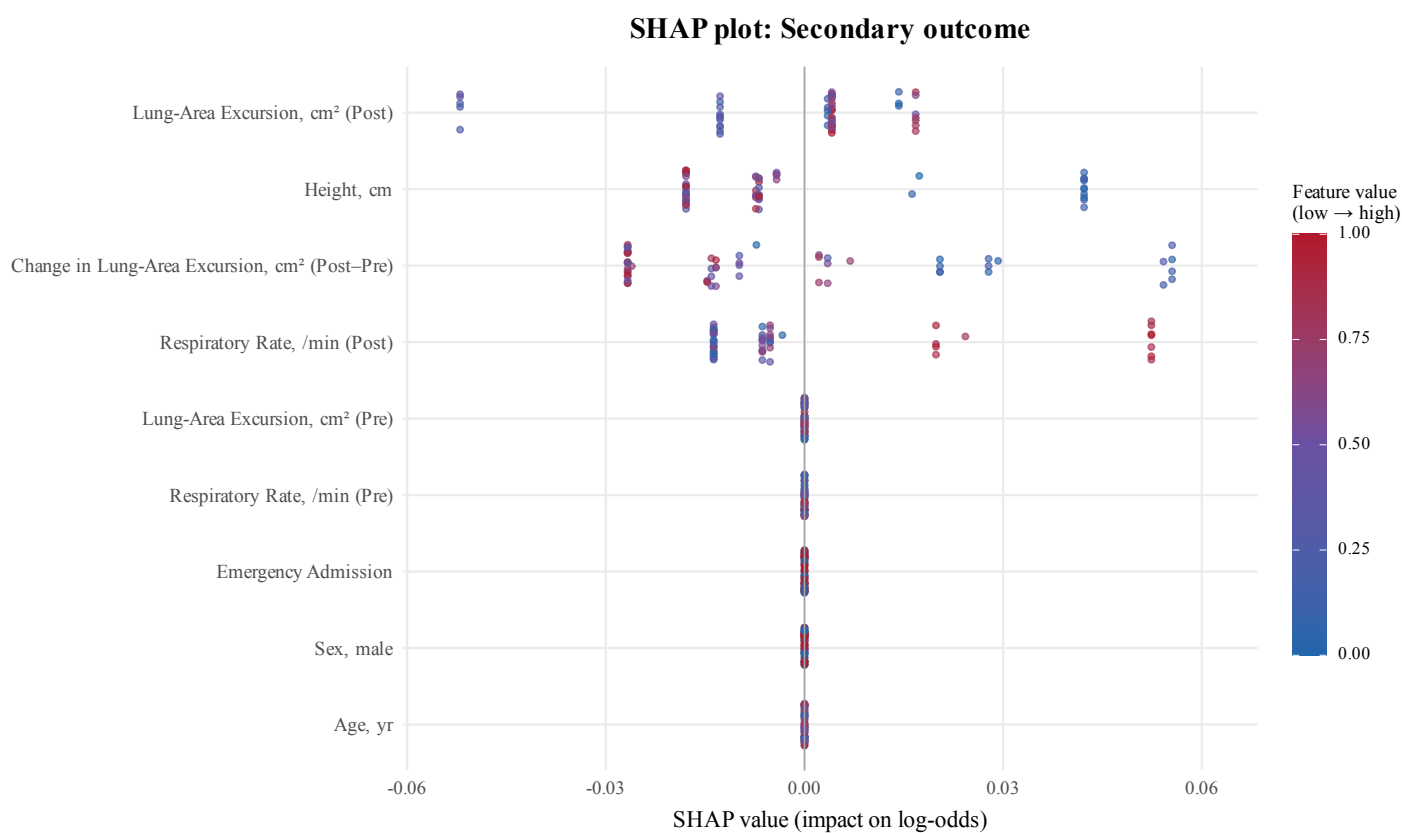

Supplement: S1 Fig — SHAP summary plot from the XGBoost model trained to predict the secondary respiratory support/oxygenation outcome, defined as NPPV/NHF use or post-extubation PaO₂/FIO₂ ≤ 300. The model used the same predictor set and hyperparameter structure as the primary XGBoost analysis, with the outcome replaced by the secondary outcome. Each point represents one patient. The x-axis shows the SHAP value, indicating each feature’s impact on the model output on the log-odds scale. Dot color reflects the feature value, with blue indicating lower values and red indicating higher values. Variables are ordered according to Gain-based feature importance in this secondary-outcome model. NPPV, noninvasive positive-pressure ventilation; NHF, nasal high-flow oxygen therapy; SHAP, Shapley additive explanations; PaO₂/FIO₂, ratio of arterial oxygen partial pressure to fractional inspired oxygen. (PDF) [file pone.0352029.s005.pdf]
